# P-994. Cellular and humoral immunological profile of Human T-Lymphotropic Virus type I (HTLV-1) in a pediatric case series

**DOI:** 10.1093/ofid/ofae631.1184

**Published:** 2025-01-29

**Authors:** Juan P Rojas, Diana Donneys, Ingara James

**Affiliations:** Valle University, Libre University, CALI, Valle del Cauca, Colombia; Libre University, CALI, Valle del Cauca, Colombia; Libre University, CALI, Valle del Cauca, Colombia

## Abstract

**Background:**

Human T- lymphotropic Virus type 1 (HTLV-1) is a powerful oncovirus. In South America there is a high incidence of cases in countries such as Brazil, Peru and Colombia, mainly affecting the indigenous and Afro-descendant population. In children, the main clinical findings are dermatological manifestations followed by lung disease, opportunistic coinfections, and autoinflammatory disorders. The main target of HTLV-1 is CD4+ T lymphocytes, which are the regulators of the acquired immune response, but It’s also capable of infecting CD8+ T lymphocytes, B cells, dendritic cells and synovial cells.

The purpose of this study is to describe the humoral and cellular immunological profile in pediatric patients diagnosed with HTLV-1 in a reference center in Cali, Colombia.

**Table 1.**
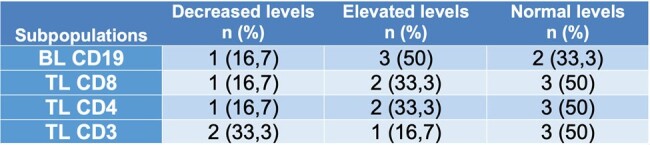
Characterization of cellular immunity in pediatric patients diagnosed with HTLV1 (n = 6) BL: B lymphocyte, TL: T lymphocyte Source: Self elaboration

**Methods:**

This is an observational, descriptive, retrospective cross-sectional study carried out between January 2017 and March 2021.

**Table 2.**
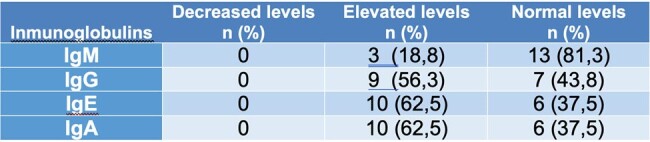
Characterization of humoral immunity in pediatric patients diagnosed with HTLV1 (n = 16)

**Results:**

Nineteen patients were included in this study with a median age at diagnosis of 94 months, most of them were schoolchildren (52.6%), 50% of patients had elevated B lymphocytes (CD19), and 50% of them had normal range cytotoxic T lymphocytes (CD8+) and helper T lymphocytes (CD4) (Table 1 and graph 1). None of the patients had decreases in serum immunoglobulins (IgM, IgG, IgE and IgA) (Table 2 and graph 2)

**Graph 1.**
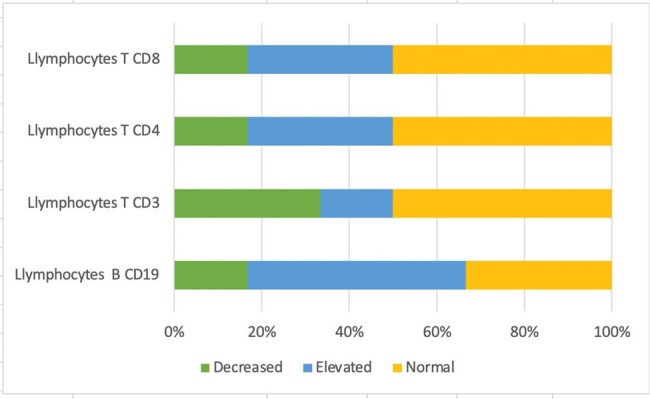
Cellular immunological profile (n=6)

**Conclusion:**

HTLV-1 immunosuppression is not mediated by low levels of lymphocyte subpopulations in the pediatric population.

**Graph 2.**
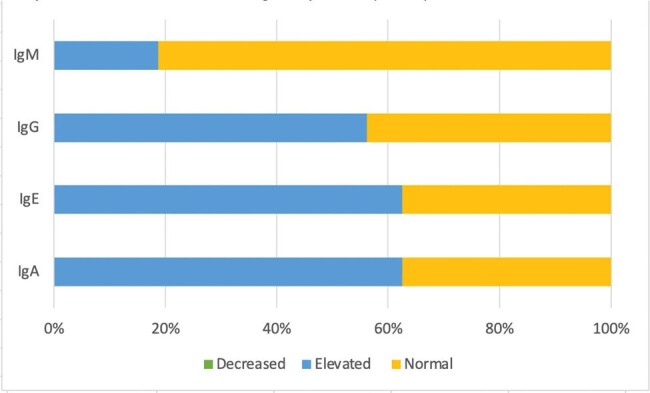
Humoral immunological profile (n=16)

**Disclosures:**

**All Authors**: No reported disclosures

